# Does Masticatory Ability Contribute to Nutritional Status in Older Individuals?

**DOI:** 10.3390/ijerph17207373

**Published:** 2020-10-09

**Authors:** Keiko Fujimoto, Hideki Suito, Kan Nagao, Tetsuo Ichikawa

**Affiliations:** 1 Department of Prosthodontics and Oral Rehabilitation, Graduate School of Biomedical Sciences, Tokushima University, Tokushima 770-8504, Japan; fujimoto.keiko@tokushima-u.ac.jp (K.F.); kan@tokushima-u.ac.jp (K.N.); 2 Department of Oral and Maxillofacial Radiology, Graduate School of Dentistry, Kyushu University, Fukuoka 812-8582, Japan; h-suito@rad.dent.kyushu-u.ac.jp

**Keywords:** nutritional status, body mass index, mastication, oral factor, masticatory efficiency, older individuals

## Abstract

Mastication plays a primary role in the process of eating. Hence, compromised masticatory ability may affect the nutrition and quality of life, which are particularly important concerns among older individuals. It remains unclear how is the masticatory ability assessed regarding the nutritional status. We examined the effect of various oral factors on three masticatory ability tests conducted among older individuals. A total of 100 older individuals were enrolled in this study. Body mass index (BMI) as an indicator of nutritional status; and age, sex, and the number of occlusal and molar occlusal supports as clinical attributes were recorded. Three masticatory ability tests (masticatory efficiency, masticatory score, and satisfaction with mastication) were conducted, and tongue pressure, cheek pressure, and occlusal force were assessed as oral functions. A significant but weak correlation was found between masticatory efficiency and the masticatory score, but not between masticatory efficiency and satisfaction score. Objective masticatory efficiency was strongly associated with objective oral factors, whereas subjective assessments of masticatory ability (masticatory score and satisfaction score) were not. Furthermore, BMI was significantly associated with subjective assessments of masticatory ability but not with objective masticatory efficiency. Both subjective and objective assessments of masticatory ability, along with considerations of nutritional formulations, are required for the maintenance and improvement of nutritional status in older individuals.

## 1. Introduction

Individuals who are overweight and obese are at an increased risk for many diseases and health conditions, and there is a direct relationship between high body mass index (BMI) and all-cause mortality [1,2,3]. Conversely, undernutrition, as indicated by a low BMI, is also associated with an increased risk of all-cause mortality; this is of particular concern among frail older adults [4,5]. Undernutrition has a varied etiology, of which impaired masticatory ability is the primary focus in the field of dentistry. Impaired masticatory ability may cause difficulties in eating, which in turn can affect the quality of life, increase the risk of undernutrition, and lead to the need for nursing care [6,7,8].

It remains unclear how masticatory ability affects the nutritional status. The relationship of subjective satisfaction with masticatory ability and objective masticatory ability (assessed via masticatory efficiency tests) in older individuals is inconclusive, with previous studies showing either a significant positive correlation [9] or the lack of a correlation [10]. In our own clinical observations, we have found that patients may be subjectively satisfied with their masticatory ability despite having a low masticatory efficiency; conversely, other patients may be dissatisfied despite having a high masticatory efficiency. While subjective self-assessments are not always in agreement with objective measurements [11,12], it is essential to elucidate the extent to which either assessment may be used for nutrition-related guidance in older individuals.

While it is well-known that mastication plays a primary role in the process of eating and that oral factors affect the masticatory ability in healthy adults, the effects in older individuals remain unclear. Some studies have suggested that masticatory ability affected by occlusal force and other occlusal factors [13,14] and also by other oral factors such as the strength and functional capacity of the tongue, lip, and cheek muscles [15,16]. However, the relationships between these factors and subjective assessments of masticatory ability have hitherto not been investigated with respect to nutritional status.

Hence, the purpose of this study was to compare the effects of various oral factors on three masticatory ability tests conducted among older individuals, with the overarching aim of determining the objective and subjective components required to guide the maintenance and improvement of nutritional status in older adults. The hypothesis was that low masticatory ability results in low nutritional status in older individuals.

## 2. Materials and Methods

### 2.1. Participants

Consecutive elderly patients reporting for regular maintenance treatment were recruited over a five-month period from the outpatient clinic of the oral rehabilitation division at Tokushima University Hospital (Tokushima, Japan).

The inclusion criteria comprised the following: (1) age over 65 years and (2) absence of a prior history of cerebrovascular disease, neurodegenerative disease, dementia, malignant tumors, and other diseases that can cause eating difficulties or dysphagia. The study was conducted in accordance with the Declaration of Helsinki and was approved by the Ethics Committee of the Tokushima University Hospital (approval number: 2225). All participants signed a written informed consent form prior to study participation.

### 2.2. Nutrition Status

Body height and weight were self-reported in participant interviews and were subsequently used to calculate the BMI for assessing the nutritional status.

### 2.3. Basic Clinical Assessments

All measurements were carried out by the same examiner (K.F.) to eliminate inter-rater variability. Prior to the commencement of the study, the examiner was trained and calibrated in performing the clinical measurements.

The age, sex, number of occlusal supports (OSN), and number of molar occlusal supports (MOSN) were recorded. The OSN and MOSN were documented during an oral examination. The OSN were obtained by counting the number of occluding tooth pairs (values ranging from 0 to 14). The MOSN were determined by counting the number of occluding molar pairs (values ranging from 0 to 8).

### 2.4. Assessments of Masticatory Ability

Subjective masticatory ability was assessed using two scales: (1) a masticatory score and (2) a conventional visual analog scale (VAS) score that evaluated satisfaction with mastication. The masticatory score was assessed using a questionnaire, which classified 25 food items into five categories based on ease of mastication for these items. Participants were asked to rate their ability to eat each food item, and the masticatory score was calculated as shown in Table 1 [17,18].

The VAS score for satisfaction with mastication consisted of a straight horizontal line, 100 mm in length, beginning with “cannot chew at all” (0 mm) and ending with “can chew very well” (100 mm). Participants marked on the line with an “X” after being asked the following question: “Can you chew well?”

**Figure 1:**
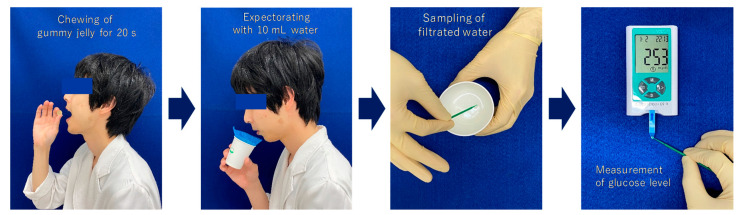
Protocol for the masticatory efficiency test.

Objective masticatory efficiency was assessed using a masticatory ability testing system (Gluco Sensor GS-II, GC Co., Tokyo, Japan) [19]. Participants were asked to chew 2 g of gummy jelly for 20 s on their habitual chewing side and then to gently expectorate it with 10 mL of water. The glucose level in the eluted filtrate is reflective of the masticatory efficiency and the glucose concentration was measured using the device mentioned above (Figure 1).

### 2.5. Assessments of Oral Muscle Strength: Tongue Pressure, Cheek Pressure, and Occlusal Force

**Figure 2:**
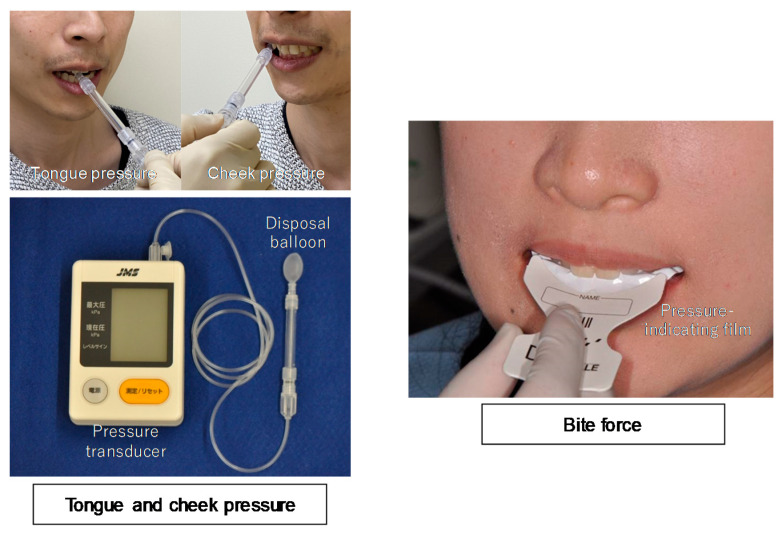
Description of tongue pressure, cheek pressure, and bite force measurements.

A device previously designed to measure tongue pressure (JMS Tongue Pressure Measuring Instrument, JMS Co., Hiroshima, Japan) was used to determine both tongue and cheek pressures (Figure 2) [20]. For the determination of tongue pressure, a disposable balloon probe was inserted into the participant’s mouth, and the participant was instructed to hold the probe gently with the front teeth. The participants were instructed to press their tongue against the pressure-sensing portion of the probe with maximum force for 7 s. For the determination of cheek pressure, the balloon probe was inserted between the right buccal mucosa and buccal surfaces of the upper and lower first molars, with the teeth in occlusion. Patients were instructed to purse their lips and press their cheek against the pressure-sensing portion of the probe with maximum force for 7 s. Both tongue and cheek pressures were measured three times, and the mean value was taken as the representative value.

Occlusal force was measured using a pressure-indicating film (Dental Prescale 50H, GC Co., Tokyo, Japan) and analyzed using an Occluser 709 (GC Co., Tokyo, Japan) (Figure 2) [21]. The film was placed between the maxillary and mandibular teeth, and the participants were instructed to bite with the maximum force for 3 s. The occlusal force was measured three times, and the maximum value was taken as the representative value.

### 2.6. Statistical Analyses

All hypotheses were specified prior to data collection. Before the pre-specified analyses were conducted, the participants were categorized into the following two groups based on the median of each masticatory ability test (masticatory efficiency, masticatory score, and VAS satisfaction score): the upper-category group (UG) and lower-category group (LG). Each reciprocal relationship between the three types of masticatory ability tests was examined using the Spearman’s correlation coefficient test. The differences in each masticatory ability test between the UG and LG were analyzed using the chi-squared test and Mann–Whitney U test.

A univariate logistic regression (ULR) analysis was performed using the forced-entry method to determine the association between the masticatory ability test score (dependent variable) and potential explanatory variables. A multivariate logistic regression (MLR) analysis was performed to identify interactions between variables, and to consider these for further analysis. Variables were selected using the step-down procedure and likelihood ratio test.

Prior to the above tests, the Shapiro-Wilk test was used to determine whether the data followed a normal distribution. The interclass correlation coefficient was also calculated to determine the reliability of the measurements. The significance level for all tests was set at *p* = 0.05. SPSS ver. 24.0 for Mac (IBM Co., Tokyo, Japan) was used for all statistical analyses.

## 3. Results

One hundred participants (53 males and 47 females; mean age, 75.3 ± 6.5 years) were enrolled in this study. The means and standard deviations (SDs) for masticatory efficiency, masticatory score, and VAS satisfaction score were 137.7 ± 57.6, 89.1 ± 15.7, and 76.0 ± 19.5, respectively. The median values for the categorizations of the UG and LG from the regression analysis are shown in Table 2.

**Figure 3:**
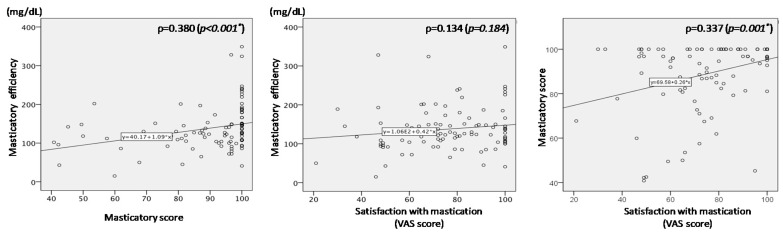
Reciprocal relationships between the three masticatory ability tests. * *p* < 0.05.

Figure 3 shows the relationships between each test pair among the three masticatory ability tests. Significant but weak correlations were observed between masticatory efficiency and masticatory score (*p* = 0.380), and between masticatory score and VAS satisfaction score (*p* = 0.337). No significant correlation (*p* = 0.184) was observed between masticatory efficiency and VAS satisfaction score.

Table 3 shows the means and SDs of measurement factors in the two groups for each masticatory ability test. The distributions of sex and age were not different between the two groups. Overall, measurement values in the UG were better than those in the LG; cheek pressure was an exception.

Table 4 shows the results of the logistic regression analysis. All correlation coefficients between each parameter were less than 0.9, and the interclass correlation coefficients for all parameters were greater than 0.8. Acceptable reproducibility of the measurements was confirmed.

The associations of masticatory efficiency with the OSN, MOSN, tongue pressure, and occlusal force were determined via ULR analysis. The MOSN and tongue pressure were found to be independent predictors of masticatory efficiency in the final MLR model. The goodness of fit of the regression model was determined using the Hosmer–Lemeshow test (*p* = 0.545), and the percentage of correct classifications was 70%.

The associations of masticatory score with the OSN, MOSN, BMI, and occlusal force were determined via ULR analysis. The OSN, cheek pressure, and BMI were found to be independent predictors of masticatory score in the final MLR model. The goodness of fit of the regression model was determined using the Hosmer–Lemeshow test (*p* = 0.698), and the percentage of correct classifications was 71%.

The associations of VAS satisfaction score with the OSN, MOSN, and BMI were determined via ULR analysis. Both age and BMI were found to be independent predictors of VAS satisfaction score in the final MLR model. The goodness of fit of the regression model was determined using the Hosmer–Lemeshow test (*p* = 0.606), and the percentage of correct classifications was 68%. 

## 4. Discussion 

Undernutrition is a major factor in the deterioration of health status, especially among frail elderly individuals [22,23,24]. The food intake factors that result in undernutrition can be influenced by the following factors: masticatory ability and eating manner, environment/ability to prepare foods, food preference and literacy [25,26,27,28]. While nutritional status will be mainly dependent on food preference and food literacy in younger individuals, masticatory ability and satisfaction with mastication assume a greater importance in older individuals [29]. In this study, we aimed to determine the most appropriate components that should be evaluated in masticatory assessments for older individuals, in order to improve the nutritional status.

In this study, we compared three masticatory ability tests among older individuals; no such comparisons have been made to date. The validity of the objective masticatory efficiency test has been previously demonstrated in studies utilizing the conventional sieve method as a gold standard [19]. The masticatory score is considered a quasi-subjective evaluation, despite being a quantitative form of assessment. This is because food intake standards vary by country; thus, every country uses its own specific food intake questionnaire. In Japan, the masticatory score described has been previously validated using the conventional sieve method [17] and is currently widely used in Japan. The evaluation of satisfaction with mastication using the VAS is a completely subjective assessment. Participants were divided into two groups (UG and LG) according to the median scores obtained in the three masticatory ability tests. The median value was selected as the threshold because the data of masticatory ability tests had distribution biases. Moreover, the meaning of the cut-off values was unclear in the three tests.

In the comparisons among the three masticatory ability tests, significant but weak correlations were found between masticatory efficiency and masticatory score, and between masticatory score and VAS satisfaction score; however, no significant correlation was found between masticatory efficiency and VAS satisfaction score. Thus, the purely subjective assessment (VAS satisfaction) had a poorer correlation to the purely objective assessment (masticatory efficiency), compared with the partially subjective assessment (masticatory score).

The significant explanatory factors were also found to differ for each masticatory ability test. With regard to objective masticatory efficiency, significant associations were found for a number of occlusal and oral factors in the ULR analysis; MOSN and tongue pressure remained significant in the final MLR model. Masticatory efficiency refers to the ability to achieve an adequate degree of comminution of food and is dependent on two major factors: the crushing of the food bolus on the occlusal table and food bolus formation via tongue movement. Therefore, it was not unexpected that both the MOSN and tongue pressure were significant predictors of masticatory efficiency.

With regard to masticatory score, occlusal force and OSN were found to be significant predictors in the ULR analysis; BMI, OSN, and cheek pressure were determined to be significant factors in the final MLR model. Higher masticatory scores were significantly associated with lower cheek pressures. Measuring tongue and cheek pressures was difficult in older participants, and the best performance was not always obtained for these measurements. As a higher cheek pressure is generally preferable for mastication, the reasons for this result remain unclear, despite taking into account the inherent errors in the measurements. In contrast to masticatory efficiency, the influence of oral factors on masticatory score was lower.

No oral factors were found to be significantly associated with VAS satisfaction score in the ULR analysis. Both BMI and age were determined to be significant predictors in the final MLR model. Thus, objective oral factors had a smaller influence on subjective, as opposed to objective, assessments of masticatory ability. VAS satisfaction score decreased with age. Furthermore, it is notable that BMI was found to be significantly associated with the two subjective assessments of masticatory ability (masticatory and VAS satisfaction scores), but not the objective masticatory efficiency test and other oral factors. A possible explanation may be that older individuals adopt strategies including choosing foods with a softer texture, consuming liquid foods e.g., soups, cutting their food into smaller pieces, and cooking foods to make them easier to eat, according to their individual masticatory ability. An urgent concern in older individuals, especially in those with frailty and geriatric syndromes, is undernutrition, which confers a high risk of mortality. Previous studies have reported that despite undergoing implant treatment and demonstrating improvements in chewing function, patients may not experience changes in dietary intake, BMI, or blood markers [30,31,32]. It is also reported that oral physiology and anatomy only explain the variations in oral processing behavior to a limited extent [33]. Therefore, it is apparent that both subjective and objective assessments of masticatory performance are required to facilitate the improvements in nutritional status of older individuals.

This study has four major limitations. First, BMI was used as an assessment of nutritional status because BMI is an easy and non-invasive indicator, and is readily available for significant clinical studies [1,2,4,5,25]. However, BMI is just one aspect of nutritional status and is influenced by various lifestyle factors such as physical activity, diet, and general health/disease status). More accurate evaluation of nutritional status could include other tests, such as a mini nutritional assessment (MNA^®^), body composition assessment using, for example, bioelectrical impedance analysis, indicators of gut function, and blood chemistry [34,35,36]. Additionally, BMI calculations in this study were based on self-reported weight and height, and these were not actually measured. As all participants had a regular physical check-up, the data were considered to be reliable; however, there may have been some bias. Second, assessments of participants’ prostheses were not carried out in this study, as the priority was the investigation of oral factors, such as tongue pressure; therefore, the evaluations of masticatory ability may have been affected by the use of existing prostheses. Third, the study lacked statistical power due to the small number of participants. Nevertheless, the number of investigated variables was reduced accordingly, and the same results were obtained in the statistical analyses. Thus, the sample size employed in this study was sufficient to draw conclusions with respect to the primary outcomes. Fourth, the participants in this study were outpatients undergoing regular maintenance at a university hospital; therefore, they were not representative of the general elderly population in Japan.

## 5. Conclusions

In conclusion, objective masticatory efficiency was found to be dependent on oral factors. This is in contrast to subjective assessments of masticatory ability, including masticatory score and VAS satisfaction score, which were dependent on the BMI and not associated with the tested oral factors, with the exception of the MOSN. 

The hypothesis could not be fully accepted, and both objective and subjective assessments of masticatory ability, along with considerations of nutritional formulations, will be required for the maintenance and improvement of nutritional status in older individuals.

## Figures and Tables

**Table 1:** The 25 food items and formula for calculating the masticatory score.

Food Group	I	II	III	IV	V
Foods	□Bananas □Boiled cabbage □Boiled carrots □Boiled taro □Boiled onions	□Strawberries □Ham □Boiled fish paste □Konnyaku □Boiled kombu	□Fried chicken □Roast chicken □Apples □Pickled eggplants □Raw cabbage	□Roast pork □Pickled radishes □Rice cakes □Peanuts □Slice draw cuttlefish	□Raw carrots □Takuwan □Vinegared octopus □Raw abalone □Dried cuttlefish
Mean score	A	B	C	D	E

□ Assessment point: [2] easily eaten, [1] eaten in difficulty, [0] cannot be eaten. A–E indicate the mean score of the assessment points in the five groups (I–V). Masticatory score = [*A* + 1.06 ∗ *B* + 1.22 ∗ *C* + 1.39 ∗ *D* + 2.23 ∗ *E*]/13.8 × 100.

**Table 2:** Regression analysis categorizations for the three masticatory ability assessments.

Groups	Masticatory Efficiency Test [mg/dL]	Masticatory Score	Satisfaction with Mastication (VAS Score)
Upper-category group (UG)	≥126.0	≥96.7	≥77.0
Lower-category group (LG)	<126.0	<96.7	<77.0
Mean and SD	137.7 ± 57.6	89.1 ± 15.7	76.0 ± 19.5

**Table 3:** Differences in measurement factors between the upper and lower category groups in assessments of masticatory ability.

Measurement Factors	Total *n* = 100	Masticatory Efficiency			Masticatory Score		Satisfaction with Mastication (VAS Score)			
		LG	UG	*p*-Value	LG	UG	*p*-Value	LG	UG	*p*-Value
		*n* = 51	*n* = 49		*n* = 50	*n* = 50		*n* = 51	*n* = 49	
Nutritional status: BMI (kg/m^2^)	22.6 ± 3.3	22.5 ± 3.0	22.7 ± 3.5	0.634	21.8 ± 2.7	23.3 ± 3.6	0.013*	21.6 ± 2.9	23.6 ± 3.3	0.001 *
Basic attributes										
Sex (male:female)	53:47	29:22	24:25	0.430 ^a^	25:25	28:22	0.548 ^a^	26:25	27:22	0.680 ^a^
Age	75.3 ± 6.5	75.9 ± 6.7	74.6 ± 6.2	0.335	76.4 ± 6.5	74.2 ± 6.2	0.111	76.5 ± 6.5	74.0 ± 6.3	0.064
Occlusal supports										
Occlusal supports (unit)	4.1 ± 5.0	2.4 ± 3.5	5.9 ± 5.7	0.003 *	2.3 ± 3.9	5.9 ± 5.4	0.000 *	3.0 ± 4.2	5.3 ± 5.5	0.059
Molar occlusal supports (unit)	1.7 ± 2.7	0.63 ± 1.6	2.7 ± 3.2	0.001 *	0.8 ± 1.9	2.5 ± 3.1	0.003 *	1.0 ± 2.2	2.3 ± 3.1	0.027 *
Gnathological muscle strength										
Tongue pressure (kPa)	27.8 ± 8.4	25.7 ± 8.3	30.0 ± 8.1	0.017 *	27.2 ± 7.4	28.4 ± 9.4	0.282	27.0 ± 8.9	28.6 ± 7.8	0.370
Cheek pressure (kPa)	16.4 ± 4.0	16.0 ± 4.4	16.9 ± 3.4	0.202	17.2 ± 3.6	15.7 ± 4.2	0.110	16.4 ± 4.4	16.5 ± 3.4	0.983
Occlusal force (N)	274.7 ± 212.4	215.1 ± 183.0	336.9 ± 224.6	0.000 *	229.7 ± 204.9	319.8 ± 212.1	0.003 *	266.8 ± 209.8	283.0 ± 217.0	0.617

Mean ± standard deviation; * *p* < 0.05 [^a^ chi-square test; ^no mark^ Mann–Whitney U-test].

**Table 4:** Regression analysis of the association between oral factors and masticatory ability test.

Tests	Measurement Actors	Univariate Logistic Regression Analysis (ULR)				Multivariate Logistic Regression Analysis (MLR)			
		Odds Ratio	95% CI		*p*-Value	Odds Ratio	95% CI		*p*-Value
			Lower Limit	Upper Limit			Lower Limit	Upper Limit	
Masticatory efficiency	Nutritional status: BMI (kg/m^2^)	1.023	0.907	1.155	0.708				
	Basic attributes								
	Sex	0.967	0.909	1.029	0.288				
	Age	1.373	0.624	3.019	0.430				
	Occlusal supports								
	Occlusal supports (unit)	1.169	1.066	1.282	0.001 *				
	Molar occlusal supports (unit)	1.430	1.167	1.753	0.001 *	1.464	1.178	1.820	0.001 *
	Oral muscle strength								
	Tongue pressure (kPa)	1.069	1.015	1.127	0.012 *	1.077	1.018	1.140	0.010 *
	Cheek pressure (kPa)	1.056	0.954	1.168	0.294				
	Occlusal force (N)	1.003	1.001	1.006	0.007 *				
Masticatory score	Nutritional status: BMI (kg/m^2^)	1.161	1.016	1.326	0.028 *	1.228	1.059	1.425	0.007 *
	Basic attributes								
	Sex	0.948	0.890	1.009	0.093				
	Age	0.786	0.358	1.726	0.548				
	Occlusal supports								
	Occlusal supports (unit)	1.178	1.072	1.294	0.001 *	1.199	1.084	1.328	0.000 *
	Molar occlusal supports (unit)	1.300	1.089	1.553	0.004 *				
	Oral muscle strength								
	Tongue pressure (kPa)	1.017	0.970	1.067	0.473				
	Cheek pressure (kPa)	0.908	0.818	1.009	0.072	0.854	0.757	0.964	0.011 *
	Occlusal force (N)	1.002	1.002	1.005	0.042 *				
Satisfaction with mastication(VAS score)	Nutritional status: BMI (kg/m^2^)	1.243	1.074	1.437	0.003 *	1.274	1.094	1.484	0.002 *
	Basic attributes								
	Sex	0.847	0.386	1.860	0.680				
	Age	0.942	0.884	1.003	0.064	0.925	0.863	0.991	0.027 *
	Occlusal supports								
	Occlusal supports (unit)	1.099	1.011	1.195	0.027 *				
	Molar occlusal supports (unit)	1.203	1.025	1.411	0.023 *				
	Oral muscle strength								
	Tongue pressure (kPa)	1.024	0.976	1.074	0.331				
	Cheek pressure (kPa)	1.010	0.914	1.115	0.848				
	Occlusal force (N)	1.000	0.998	1.001	0.703				

* *p* < 0.05.
